# Metabolic and immunological phenotype of rare lipomatoses: Dercum’s disease and Roch-Leri mesosomatic lipomatosis

**DOI:** 10.1186/s13023-021-01920-3

**Published:** 2021-06-29

**Authors:** Madleen Lemaitre, Benjamin Chevalier, Arnaud Jannin, Kristell Le Mapihan, Samuel Boury, Georges Lion, Myriam Labalette, Marie-Christine Vantyghem

**Affiliations:** 1 Endocrinology, Diabetology and Metabolism, CHU Lille, 59000 Lille, France; 2 Nuclear Medicine Department, CHU Lille, 59000 Lille, France; 3 Institute of Immunology, CHU Lille, 59000 Lille, France; 4 Inserm U1190, 59000 Lille, France; 5 Univ. Lille, 59000 Lille, France

**Keywords:** Lipodystrophy, Dercum’s disease, Roch-Leri, Lipomatosis, Basophil, Natural killer, T lymphocyte, CD4 (cluster differentiation 4), CD3, CD8

## Abstract

**Context:**

Dercum’s disease (DD) and Roch-Leri mesosomatic lipomatosis (LMS) are rare and poorly characterized diseases. The clinical presentation combines multiple lipomas, painful in DD in contrast with LMS, without lipoatrophy.

**Objective:**

To identify any specific metabolic and immune phenotype of DD and LMS.

**Design and patients:**

This monocentric retrospective study included 46 patients: 9 DD, 11 LMS, 18 lean and 8 obese controls. Metabolic and immunohematological characteristics of each group were compared.

**Results:**

The median age of the patients was similar in the 3 groups (31 years). The number of women, and of basophils, and CD3^+^, CD4^+^ and CD8^+^ T lymphocytes was significantly higher in the DD versus the LMS group, without any difference of the metabolic parameters. Weight, BMI, blood pressure, gamma-GT, leptin, fasting insulin and C-peptide levels, fat mass percentage, and intra/total abdominal fat ratio were significantly higher in each lipomatosis group compared with the lean group. Compared with the lean group, the DD group had significantly higher fasting blood glucose, LDL-cholesterol, platelets, leukocytes, basophils, and a lower NK cell count, whereas the LMS group had a significantly lower rate of CD3, CD4, and CD8 lymphocytes. Compared with the obese controls, basophils remained higher in DD and T lymphocytes subpopulations lower in LMS groups.

**Conclusion:**

DD and LMS show a common background of obesity and metabolic phenotype, but a distinct immunohematological profile characterized by a higher number of basophils in DD patients, an inflammatory profile that could contribute to pain. T lymphocyte depletion was present in LMS. These findings could offer specific therapeutic opportunities, especially for painful DD.

**Supplementary Information:**

The online version contains supplementary material available at 10.1186/s13023-021-01920-3.

## Introduction

Lipodystrophy syndromes are rare diseases characterized by a limited capacity of subcutaneous adipose tissue to store triglycerides, which results in metabolic abnormalities such as insulin resistance, hypertriglyceridemia, fatty liver disease and polycystic ovary syndrome. Apart from these syndromes that are usually associated with partial or generalized lipoatrophy, lipomatosis is defined by the presence of multiple lipomas on the body, without lipoatrophy [1–3].

Different entities accompanied by multiple lipomas have been described, including:

- Syndromic lipomatosis, such as those encountered in type 1 Multiple Endocrine Neoplasia or certain genetically determined multiple lipomatosis [4];
- Multiple symmetric lipomatosis, most often linked to alcohol (Madelung or Launois-Bensaude disease);
- Dercum's disease, also known as *adiposis dolorosa* or Ander’s syndrome;
- Mesosomatic lipomatosis (LMS), also called Roch-Leri lipomatosis [5];
- Hibernomas, epidural lipomatosis and familial angiolipomatosis.

Of these lipomatous syndromes, LMS and Dercum's disease, the diagnosis of which is clinical, remain poorly described. Only isolated clinical cases or small studies aiming to report surgical treatment are mentioned in the literature [6–9].

Dercum's disease* is a very rare disease characterized by multiple, painful subcutaneous lipomas, occurring mainly on the trunk, and the proximal part of the arms and legs [10, 11]. The disease is often associated with obesity, asthenia and various neurological disorders, including depression and epilepsy [12]. The pathophysiology of Dercum's disease remains unknown although various mechanisms have been suggested, such as autoimmunity, alterations in the metabolism of fatty acids, carbohydrates or hormones, previous infections [13] or abnormal lymphatic tissue [14]. The majority of reported cases are sporadic, but a few apparently autosomal dominant familial cases have been reported [15–17]. In addition to other multiple lipoma syndromes, the differential diagnosis includes fibromyalgia and lipedema [18]. Treatment is symptomatic, mainly for analgesic purposes. Recurrence of lipomas after surgical removal is common.

Roch-Leri lipomatosis** is a disorder of adipose tissue proliferation characterized by the presence of generally painless, multiple, small lipomas, 2–5 cm in diameter, in the middle third of the body (forearms, trunk, thighs). They are easy to remove under local anaesthesia if not too numerous or confluent. No report of this partially forgotten syndrome has been available in PubMed since 1984, probably because it is usually considered harmless. Autosomal dominant cases have been reported but no gene has been identified and sporadic cases seem to be the most common [5].

These two mild disorders of Dercum’s and Roch-Leri have piqued little clinical interest until recent findings on the heterogeneity of adipose tissue, its regenerative capacity, and its regulatory role in metabolism through the secretion of hormones and inflammatory mediators [19]. A better understanding of these “benign” disorders could help to identify a currently lacking diagnostic biomarker and to better understand the mechanisms of these diseases and the pathophysiology of adipose tissue. Therefore, the aim of the present study was to determine the clinical-immunological phenotype of Dercum's disease and LMS in comparison with control subjects.

*After Dr Dercum, an American neurologist (1856–1931); **after M. Roch, a Swiss internist (1878–1967) and A. Leri, a French doctor (1875–1930).

## Patients and methods

### Study design

This retrospective study was conducted at one university hospital over a decade from 2009 to 2019. All the patients referred to the institution’s endocrinology and metabolism department with a final diagnosis of Dercum’s disease or mesosomatic lipomatosis were included and compared with 2 age and sex-matched control groups, one lean, the other obese, whose subjects had been recruited from the NCT0178428 trial. This study protocol was approved by the relevant ethics committee, and all selected subjects gave their written informed consent to participate. Thus, this case–control study included men and women aged > 18 years from the following groups:

- Patients with Dercum’s disease (DD group).
- Patients with Roch-Leri mesosomatic lipomatosis (LMS group).
- Normal weight control subjects (BMI (Body Mass Index) > 18 kg/m^2^ but < 25 kg/m^2^) (Lean group).
- Obese control subjects without lipoma (BMI > 25 kg/m^2^) (Obese group).

The clinical and metabolic phenotypes were compared between DD, LMS and lean groups. The immune-hematological phenotype was compared between the four groups (DD, LMS, lean and obese).

### Patients and controls

A total of 76 patients were referred for a qualitative abnormality of adipose tissue over the ten-year period. After subjecting these patients to careful clinical and routine laboratory testing, those with a clinical phenotype suggestive of Dercum’s disease or Roch-Leri-LMS were included.

Patients from the control groups were excluded if they were aged < 18 years or fulfilled any of the following criteria: creatinine > 1.5 mg/dL; active cancer; excessive alcohol consumption; coagulation disorders; active autoimmune or chronic infection, including human immunodeficiency virus (HIV) and hepatitis C; treatment that might interfere with metabolic function, including estrogens and analogues (contraceptive pill, tamoxifen, etc.); and other medico-legal conditions (psychiatric disease; pregnant or breastfeeding women, etc.).

Clinical and biological data were collected from the patients’ medical files at the time of assessment or, for the control groups, from the NCT0178428 database.

### Outcomes

#### Clinical parameters

Age, sex, height and body weight, and a family history of lipomatosis, diabetes mellitus or obesity were recorded, and BMI was calculated. Hypertension, defined as blood pressure > 130/85 mmHg [20] or the use of an antihypertensive drug, was recorded, as well as the use of lipid-lowering agents (such as statins, fibrates, ezetimibe), the use of antidiabetic treatments (such as lifestyle modification, metformin or any other antidiabetic drugs, including glucagon-like peptide-1 receptor agonists and insulin); and the use of analgesics and mood stabilizers.

The number and locations of lipomas were also recorded, as well as any previous history of lipoma surgery. The encapsulated nature of lipomas and the fibrous component of adipose tissue was studied by ultrasonography.

#### Metabolic parameters

- Fasting blood glucose (FBG), liver enzymes (aspartate aminotransferase [AST], alanine aminotransferase [ALT], gamma-glutamyl transferase [GGT]), triglycerides, low-density lipoprotein (LDL) and high-density lipoprotein (HDL) cholesterol levels were measured using routine methods.
- Fasting insulin levels were measured by monoclonal immunoradiometric assay (Bi-INS-IRMA; Cisbio, Bedford, MA, USA) and fasting C-peptide using radioimmunoassay (RIA-coat C-peptide [Mallinckrodt France SARL, Paris, France], detection limit: 0.2 ng/mL).
- Leptin levels were measured by radioimmunoassay using commercial kits (Human Leptin RIA, EMD Millipore, Billerica, MA, USA). Intra- and inter-assay coefficients of variation (CVs) were < 8.5%.
- Diabetes and glucose intolerance were assessed by subjecting participants who were not already being treated for diabetes at inclusion to a 75-g oral glucose tolerance test (OGTT), which was interpreted according to the American Diabetes Association’s criteria.
- HOMA-IR (Homeostatic Model Assessment of Insulin Resistance) was calculated according to the formula: insulin (mIU/L) x glucose (mmol/L)/22.5.

#### Body composition parameters

- Body fat percentage was measured by dual-energy X-ray absorptiometry (DEXA; Lunar DPX-IQ, GE Healthcare, Chicago, IL, USA).
- Total and intra-abdominal fat, an estimation of subcutaneous and visceral fat, were measured from fat surface areas of 1-cm reconstructed slices of abdominal L4 magnetic resonance imaging (MRI).

#### Immunohematological parameters

The following were documented in the four groups:

- Personal history of immunoinflammatory disease (note that a history of autoimmune disease was an exclusion *criteria* for the control group).
- Blood count as well as platelets according to routine techniques.
- Lymphocyte immunophenotyping using flow cytometry (Navios flow cytometer, Beckman Coulter).

### Statistical analysis

Quantitative variables were expressed as medians and first and third quartiles on graphs and as medians with minimum–maximum values in tables. Inter-group comparisons were performed using the Kruskal–Wallis or Mann–Whitney U tests. Qualitative values were expressed as proportions and compared between groups using the chi-square or Fisher’s exact test according to validity conditions. Analyses were carried out with GraphPad Prism 6 software (GraphPad Software Inc., La Jolla, CA, USA). Any differences with *p* values < 0.05 were considered significant.

## Results

### Characteristics of the groups

Overall, of the 76 patients referred for suspicion of lipodystrophy syndromes, 56 were excluded, whom

- 52 for other types of lipodystrophy syndromes:
  - 29 genetically-determined among which 23 *LMNA*-related,
  - 8 cases of Launois-Bensaude,
  - 2 cases of lipedema, 2 Barraquer-Simons and 1 Lawrence syndromes,
  - 10 from unknown origin.
- four because of insufficient data or persistent diagnostic doubt.

Twenty lipomatosis cases were finally included:

- 9 patients with Dercum's disease (3 men and 6 women)
- and 11 patients with Roch-Leri lipomatosis (7 men and 4 women).

**Fig. 1:**
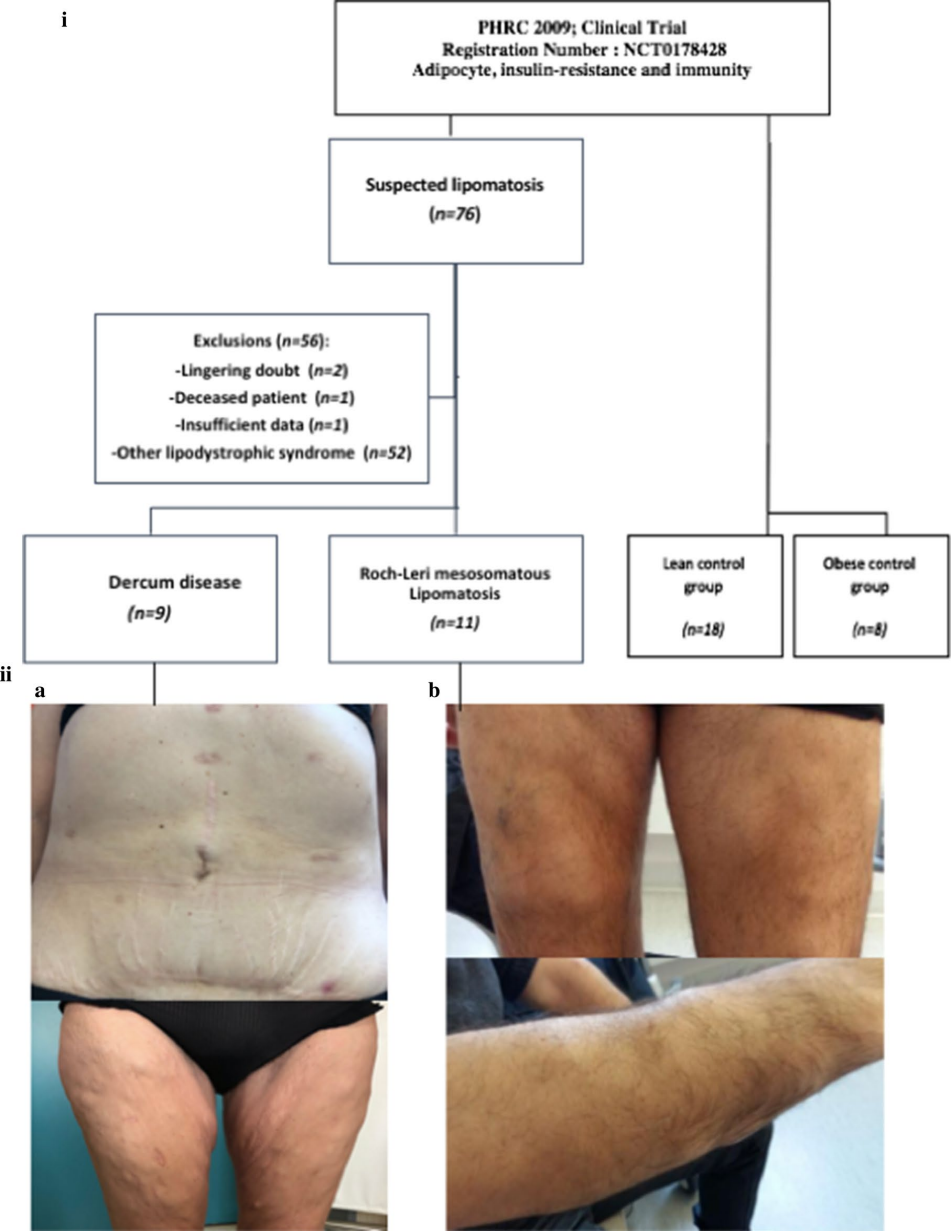
Patient enrollment flow-chart and iconography. 1-I: flow chart.1-II: Iconography of Dercum’s disease and Roch-Leri lipomatosis: on the left, patient with Dercum's disease: multiple encapsulated lipomas disseminated on the thighs associated with a pain component (a). On the right, patient with Roch-Leri LMS: multiple encapsulated lipomas, painless, disseminated on the forearms (b)

In addition, 18 lean (10 men and 8 women) and 8 obese controls without lipomas (4 men and 4 women) were included, resulting in a total of 46 patients (24 men and 22 females) (Fig. 1-I).

#### Clinical characteristics of the Dercum’s disease group

As shown in Table 1, the sex ratio was 2:1 for women and men, with a median age of 30 years and a BMI of 32.5 kg/m^2^. The lipomas were mainly distributed on the thighs (67%), back (54%) and forearms (56%). Most patients (89%) had more than 10 lipomas (Fig. 1-II). The ultrasonographic characterization showed that 88% of lipomas were encapsulated, 11% were fibrotic, and 11% were ecchymotic. Pain, which was an inclusion *criteria* for the Dercum’s group, was present in 100% of cases, with 88% having paroxysmal pain and 12% chronic pain. More than half of the patients (56%) had undergone at least one surgery, and the pathological analysis confirmed the diagnosis of lipoma in all cases. Respectively, 11%, 22% and 33% were treated for dyslipidemia, hypertension and diabetes before the diagnosis. For the pain, 33% of patients used level I and 56% level II analgesics; 36% received mood modulators (benzodiazepines and antidepressants) and 22% anti-epileptics. The proportion of immune function disorders in the personal history of the Dercum’s patients was 22%. Eleven percent of the patients had a family history of first- degree lipomatosis.

**Table 1:** Main phenotypic characteristics of patients belonging to the Dercum’s disease and Roch Leri lipomatosis (LMS) groups

Characteristics at diagnosis	Dercum (n = 9)	LMS (n = 11)	*p*
*Clinical data*			
Sex-ratio (M/F)	0.5 (3 M/6F)	1.75 (7 M/4F)	**0.005**
Age (years)	30.8 [16–50]	31.2 [18–68]	0.69
Weight (kg)	89 [58–98]	100 [55–142]	0.42
BMI (kg/m^2^)	32 [27–59]	31 [25–35]	0.71
Number of lipomas > 10 (%; n/N)	89% (8/9)	82% (9/11)	> 0.9
Encapsulated—fibrotic—ecchymotic (%; n/N)	89–11–11% (9/9)	100–9–9% (11/11)	> 0.9
History of surgery (%; n/N)	56% (7/9)	54.5% (6/11)	> 0.9
*Lipomas sites*			
Forearm/arm (%; n/N)	56% (5/9)	82% (9/11)	**0.042**
Back	56% (5/9)	27% (3/11)	0.87
Abdomen	44% (4/9)	55% (6/11)	0.99
Thigh	67% (6/7)	73% (8/11)	> 0.99
Flank/ Lumbar fossa	44% (4/9)	36% (4/11)	> 0.99
Legs	11% (1/9)	27% (3/11)	0.80
Other	44% (4/9)	9% (1/11)	**0.048**
*Personal history*			
Treated hypertension (%; n/N)	22% (2/9)	64% (7/11)	0.33
Diabetes (%; n/N)	33% (3/9)	9% (1/11)	0.28
Treated dyslipidemia (%; n/N)	11% (1/9)	45% (5/11)	0.16
*Family history*			
History of lipomatosis	11% (1/9)	27% (3/11)	> 0.9
History of diabetes	55% (5/9)	9% (1/11)	> 0.9

The number of patients studied was indicated only when at least one data point was missing: n: number of examinations performed/ N: total number of patients in the group

*: *p* < 0.05, **: *p* < 0.01, ***: *p* < 0.001, ns: non significant;* p* > 0.05*: *p* < 0.05, **: *p* < 0.01, ***: *p* < 0.001, ns: non significant

#### Clinical characteristics of the Roch-Leri group

As displayed in Table 1, the sex-ratio showed a male predominance, and patients had a median age of 31 years and a BMI of 30.8 kg/m^2^. The painless lipomas were mainly located on the forearms (82%), thighs (73%) and abdomen (55%) (Fig. 1-II). More than 80% of patients (82%) had more than 10 lipomas, which were always encapsulated, and 11% were fibrotic. Around half of the patients (54.5%) had undergone surgery for at least one lipoma, and the pathological analysis confirmed the diagnosis of lipoma in all cases. Respectively, 45%, 65% and 0% of patients were treated for dyslipidemia, hypertension and diabetes before the diagnosis. One case of diabetes was diagnosed with the OGTT (9% of the LMS group). None of the patients received long-term analgesics or mood stabilizing medications. The proportion of immune function disorders in the personal history concerned almost half of patients (45%). Around one-third of patients (27%) had a family history of first-degree lipomatosis.

### Comparison of the Dercum’s disease and the Roch-Leri groups

#### Clinical phenotype

The sex ratio differed between the Dercum’s disease and LMS groups (*p* < 0.05), with a predominance of men (63%) in the LMS group and of women (66%) in the Dercum’s group. There was no difference between the lipomatosis groups with regard to age at the time of the first assessment, around 31 years for the two groups. The location of lipomas did not differ significantly between the two groups except for a higher number of lipomas on forearms/arms in the LMS *vs.* the Dercum’s group (82% vs. 56%; *p* < 0.05). The frequency of patients with a number of lipomas above 10 (around 80–90%) and the percentage of patients who had at least one lipoma surgery did not differ between the two groups despite the fact that lipomas were painful and required analgesics in Dercum’s disease (Table 1).

#### Metabolic phenotype

There were no differences in the lipid, liver enzyme, fasting glucose, insulin, C-peptide, HOMA-IR and leptin levels between the two groups (Table 2). Body composition parameters did not differ between the groups. The prevalence of treated hypertension, diabetes or dyslipidemia was similar (Table 1).

**Table 2:** Main metabolic characteristics of patients belonging to the Dercum’s disease and Roch Leri lipomatosis (LMS) groups compared to the lean control group

Metabolic characteristics	Dercum (n = 9)	LMS (n = 11)	Lean controls (n = 18)	p Dercum versus Lean	p LMS versus Lean	p Dercum versus LMS
*Clinical data*						
Age (years)	30.8 [16–50]	31.2 [18–68]	32.8 [19–65]	0.35	0.16	0.69
Weight (kg)	89 [58–98]	100 [55–142]	69 [49–83]	**0.01**	**0.001**	0.42
BMI (kg/m^2^)	32.5 [27–59]	31 [25–35]	22 [17–25]	**0.0005**	**0.0002**	0.71
Systolic Blood Pressure (mmHG)	130 [130–160]	140 [115–150]	115 [103–146]	**0.013**	**0.001**	0.42
Diastolic Blood Pressure (mmHG)	78.6 [60–90]	79.7 [70–95]	72.5 [68–89]	**0.04**	**0.04**	0.93
*Metabolic data*						
Triglycerides (g/L)	1.22 [0.65–9.42]	1.37 [0.74–2.63]	0.87 [0.56–2.77]	0.07	0.83	0.98
HDL-c (g/L)	0.45 [0.34–0.55]	0.48 [0.41–0.82]	0.53 [0.38–0.72]	0.17	> 0.99	0.15
LDL-c (g/L)	1.46 [0.83–2.72]	1.22 [0.91–1.87]	1.16 [0.92–1.4]	**0.03**	0.72	0.20
AST (IU/L)	27 [18–43]	25 [21–81]	22 [17–29]	0.05	0.09	> 0.9
ALT (IU/L)	26 [14–75]	28,5 [11–78]	17 [10–31]	0.05	0.09	0.72
Gamma-GT (IU/L)	83 [58–125]	74 [30–139]	18.7 [11–28]	**0.0001**	**0.0001**	0.54
Fasting blood glucose (g/L)	1 [0.92–1.33]	0.98 [0.84–1.32]	0.86 [0.77–1.01]	**0.0028**	0.057	0.27
Fasting insulin (µIU/L; n/N)	8.1 [6.3–14-4] (7/9)	7.35 [6.3–35.8](7/11)^a^	4.6 [1.2–9.2] (18/18)	**0.004**	**0.014**	0.68
Fasting C-peptide (ng/mL; n/N)	3.2 [2.46–4.33](7/9)	2.57 [1.57–5.21](7/11)^a^	1.75 [1–3.7] (18/18)	**0.016**	**0.04**	0.84
HOMA-IR (n/N)	2.67 [1.02–4.2] (7/9)	1.67 [1.4–11.6](7/11)^a^	1 [0.25–2.18](18/18)	**0.0008**	**0.02**	0.62
Leptin (ng/mL)	47.9 [6.7–224]	28.5 [13–110.8]	5.1 [4.1–16.1]	**0.003**	**0.0002**	> 0.9
*Anthropometric data*						
Fat mass estimated in DEXA (%; n/N)	37.1 [27.9–44.5](6/9)	34.3 [31.5–42.8] (8/11)	22.9[17.9–34.9] (18/18)	**0.008**	**0.004**	> 0.9
Intra/total abdominal fat ratio (n/N)	0.36 [0.20–0.98](8/9)	0.31[0.24–0.6] (5/11)	0.19[0.10–0.60] (18/18)	**0.04**	**0.03**	0.87

The number of patients studied was indicated only when at least one data point was missing: n: number of examinations performed/ N: total number of patients in the group

^a^Exclusion of diabetic patients

*: *p* < 0.05, **: *p* < 0.01, ***: *p* < 0.001, ns: non significant;* p* > 0.05*: *p* < 0.05, **: *p* < 0.01, ***: *p* < 0.001, ns: non significant

#### Immunohematological phenotype

The proportion of immune system disorders, such as vitiligo and dysthyroidism, in the Roch-Leri group (45%) tended to be higher than that observed in the Dercum’s group (22%), but the difference was not significant. Similarly, the blood cell count (hemoglobin, platelets, leukocytes) and the differential were similar between the Dercum’s and LMS groups except for the number of basophils, which was significantly higher (2.7-fold) in the Dercum’s than in the LMS group (*p* < 0.001). Also, the lymphocyte subpopulation count showed that the LMS group had a significantly lower total CD3^+^ T lymphocytes levels and CD4^+^ and CD8^+^ subpopulations compared with the Dercum’s group (*p* < 0.05) (Table 3 and Additional file 1: Figure S2).

**Table 3:** Main immunohematological characteristics of Dercum’s disease (DD) and Roch-Leri lipomatosis (LMS) groups compared to the lean control group

Characteristics at diagnosis	Dercum (n = 9)	LMS (n = 11)	Lean controls (n = 18)	p DD versus Lean	p LMS versus Lean	p DD versus LMS
*History*						
Dysimmune disorders (%; n/N)	22% (2/9)	45% (5/11)	0% (18/18)	*–*	*–*	–
*Paraclinical*						
Hemoglobin (g/dL)	14.3 [12.5–15.4]	14.6 [12–15.4]	15 [12.1–17.3]	0.07	0.41	0.46
Platelets (10^3^/mm^3^)	266 [211–310]	220 [104–255]	210 [176–338]	**0.005**	0.39	0.10
Leukocytes (10^3^/mm^3^)	7.8 [5–9.1]	5.8 [5.2–9.8]	5.4 [4.3–11.1]	**0.02**	0.49	0.19
CRP (mg/L)	3 [3–18]	3 [3–10]	3 [3, 4]	0.11	0.65	0.52
*Leukocyte differential: (/mm*^3^)						
Lymphocytes	1939 [1100–2000]	1832 [600–2300]	1950 [1600–3300]	> 0.9	0.72	0.63
Monocytes	500 [500–700]	400 [400–600]	400 [300–900]	0.21	> 0.9	0.33
Nuclear neutrophils	5200 [2600–10000]	3300 [2500–4600]	2800[1200–3260]	0.50	0.35	0.14
Eosinophils	150 [0–300]	100 [0–500]	100 [0–1600]	> 0.9	0.26	0.20
Basophils	52 [30–100]	19 [0–37]	0 [0–14]	**0.001**	0.57	**0.001**
*Lymphocytes subpopulations (n/N)*	(5/9)	(7/11)	(18/18)			
CD3+	1420 [1105–1904]	867 [513–1584]	1444[913–2607]	> 0.9	**0.009**	**0.04**
CD4+	1255 [790–1710]	636.5 [328–922]	866 [367–1653]	0.23	**0.048**	0.03
CD8+	479 [454–788]	227[140–310]	546 [284–943]	> 0.9	**0.014**	0.007
B Lymphocytes	204.5 [140–760]	227 [101–310]	211 [103–528]	> 0.9	> 0.9	0.93
NK Lymphocytes	207.5 [40–225]	210 [124–300]	292 [136–450]	**0.049**	0.31	0.53

The number of patients studied was indicated only when at least one data point was missing: n: number of examinations performed/ N: total number of patients in the group

*: *p* < 0.05, **: *p* < 0.01, ***: *p* < 0.001, ns: non significant;* p* > 0.05*: *p* < 0.05, **: *p* < 0.01, ***: *p* < 0.001, ns: non significant

### Comparison of the Dercum’s group with the lean control group

#### Metabolic characteristics

Weight (*p* < 0.05), BMI (*p* < 0.01), systolic and diastolic blood pressure (*p* < 0.05) and percentage of fat mass and intra-/total abdominal fat ratio (*p* < 0.01) were significantly higher in the Dercum’s group than in the lean control group (Table 2 and Additional file 1: Figure S2). Likewise, the levels of gamma-GT, fasting blood glucose, insulin, C-peptide, HOMA-IR, LDL-cholesterol and leptin were significantly higher in the Dercum’s group than the control group (Table 2 and Additional file 1: Figure S2). Age, triglycerides, HDL-cholesterol levels as well as liver enzymes did not differ between the Dercum’s and the lean control groups.

#### Immunohematological characteristics

The levels of platelets and circulating leukocytes were significantly higher in the Dercum’s group than in the lean control group (Table 3). The leukocyte differential (levels of lymphocytes, monocytes, nuclear neutrophils, circulating eosinophils) did not differ between the Dercum’s and the lean control groups. Only the levels of circulating basophils were significantly higher (52-fold) in the Dercum’s group compared with the lean group (*p* < 0.05). The lymphocyte subpopulation count did not differ between the Dercum’s and lean control groups except for circulating natural killer (NK) cells, which were significantly lower in the Dercum’s disease group (*p* < 0.05) compared with lean controls (Table 3 and Additional file 2: Figure S3).

### Comparison of the Roch-Leri -group with the lean control group

#### Metabolic characteristics

Weight (*p* < 0.05), BMI (*p* < 0.01), systolic and diastolic blood pressure (*p* < 0.05) and percentage of fat mass and intra-/total abdominal fat ratio (*p* < 0.01) were significantly higher in the Roch-Leri group compared with the lean control group (Table 2 and Additional file 1: Figure S2). Likewise, the levels of gamma-GT, fasting insulin, C-peptide, HOMA-IR and leptin were significantly higher in the Roch-Leri-group compared with the control group (Table 2 and Additional file 1: Figure S2). Age, and levels of fasting blood glucose, lipids and liver enzymes did not differ between the Roch-Leri group and the lean control group.

#### Immunohematological characteristics

The blood cell count and the leukocyte differential at diagnosis did not differ between the Roch-Leri group and the lean control group. The lymphocyte subpopulation count showed CD3^+^, CD4^+^, and CD8^+^ lymphocytes levels significantly lower (around 1.9-fold) in the Roch-Leri group compared with the lean control group (*p* < 0.05) (Table 3 and Additional file 2: Figure S3).

### Comparison of immuno-hematological parameters of the lipomatosis ad lean groups with the obese control group

The levels of haemoglobin, platelets, circulating leukocytes, the differential (lymphocytes, monocytes, nuclear neutrophils, circulating eosinophils) and CRP levels did not differ between the lipomatosis groups and the obese control group, except for the levels of circulating basophils which were significantly higher (2.4-fold) in the Dercum’s group compared with the obese group (*p* < 0.05). The blood cell count and differential including basophils were similar between the lean and obese control groups.

The lymphocyte subpopulation count did not differ between the Dercum’s or Roch-Leri’s groups as compared to the obese control group except for the circulating natural killer (NK) cells, which tended to be lower in the Dercum’s group than in the obese group (*p* = 0.06) (Table 4). The lymphocyte subpopulation count showed CD3^+^, CD4^+^, and CD8^+^ lymphocytes levels significantly lower only in the Roch-Leri group compared with the obese group (*p* < 0.05). The lymphocyte subpopulations were similar between the lean and the obese control groups (Table 4).

**Table 4:** Main clinical and immunohematological characteristics of Dercum’s disease (DD) and Roch-Leri lipomatosis (LMS) groups compared to an obese control group

Metabolic characteristics	Dercum(n = 9)	LMS(n = 11)	Obese controls(n = 8)	p DD versus obese	p LMS versus obese	p obese versus lean
*Clinical data*						
Sex ratio (M/W)	0.5 (3 M/6F)	1.75 (7 M/4F)	0.5 (4 M/4F)	> 0.99	> 0.99	0.09
Age (years)	30.8 [16–50]	31.2 [18–68]	46.5 [23–64]	0.44	0.21	> 0.99
BMI (kg/m^2^)	32 [27–59]	31 [25–35]	34.1 [31–43]	> 0.99	> 0.99	**0.002**
Treated hypertension (%; n/N)	22% (2/9)	64% (7/11)	38%(3/8)	> 0.99	> 0.99	0.27
Diabetes (%; n/N)	33% (3/9)	9% (1/11)	25% (2/8)	> 0.99	> 0.99	0.75
Treated dyslipidemia (%; n/N)	11% (1/9)	45% (5/11)	38% (3/8)	> 0.99	> 0.99	0.22
*Paraclinica*						
Hemoglobin (g/dL)	14.3 [12.5–15.4]	14.6 [12–15.4]	14.9[12.8–16.6]	0.47	> 0.99	> 0.99
Platelets (10^3^/mm^3^)	266 [211–310]	220 [104–255]	242 [221–283]	0.8	0.51	0.09
Leukocytes (10^3^/mm^3^)	7.8 [5–9.1]	5.8 [5.2–9.8]	6.9 [5.1–11.3]	0.97	0.74	0.06
CRP (mg/L)	3 [3–18]	3 [3–10]	6.3 [3–10]	> 0.99	> 0.99	> 0.99
*Leukocyte differential: (/mm*^3^)						
Lymphocytes	1939 [1100–2000]	1832 [600–2300]	2141 [1500–3300]	0.71	0.46	0.97
Monocytes	500 [500–700]	400`[400–600]	400 [300–700]	> 0.99	> 0.99	> 0.99
Nuclear neutrophils	5200 [2600–10000]	3300 [2500–4600]	4050 [2600–7100]	> 0.99	> 0.99	0.16
Eosinophils	150 [0–300]	100 [0–500]	188 [100–400]	> 0.99	0.35	> 0.99
Basophils	52 [30–100]	19 [0–37]	22 [0–100]	**0.03**	0.97	0.14
*Lymphocytes subpopulations (n/N)*	(5/9)	(7/11)	(8/8)			
CD3+	1420 [1105–1904]	867 [513–1584]	1688[1215–1982]	0.94	**0.02**	0.84
CD4+	1255 [790–1710]	636.5 [328–922]	979 [785–1454]	0.96	**0.01**	0.77
CD8+	479 [454–788]	227[140–310]	493 [209–992]	> 0.99	**0.02**	0.89
B Lymphocytes	204.5 [140–760]	227 [101–310]	199 [152–346]	0.73	> 0.99	> 0.99
NK Lymphocytes	207.5 [40–225]	210 [124–300]	287 [226–634]	0.06	0.31	> 0.99

The number of patients studied was indicated only when at least one data point was missing: n: number of examinations performed/ N: total number of patients in the group

*: *p* < 0.05, **: *p* < 0.01, ***: *p* < 0.001, ns: non significant;* p* > 0.05*: *p* < 0.05, **: *p* < 0.01, ***: *p* < 0.001, ns: non significant

## Discussion

The aim of this monocentric study was to improve the phenotyping of two rare forms of lipomatosis, Dercum’s disease and Roch-Leri lipomatosis. The results suggest that lipomas occur on a common background of obesity and metabolic profile in the two diseases. Interestingly, the immunohematological profiles were distinct.

The first limitations of the present analysis are the retrospective design and the clinical diagnosis of these 2 rare lipomatosis since no specific biomarker is known. In contrast, this work is the first comparative study to include well-defined lean and obese control groups with about 20 cases of lipomatosis, and the first to compare the immunohematological profile, albeit the number of patients included is low.

### Comparison of the Dercum’s disease and the Roch-Leri groups

Clinically, the female predominance of Dercum’s disease and the male predominance of Roch-Leri lipomatosis are in concordance with the literature and suggest gender difference in the expression of the disease. The age of occurrence of the two diseases was very close around 30 years old, which is similar to that mentioned in literature for LMS [5]. In contrast, it is much younger for Dercum’s disease, which is usually considered a postmenopausal illness [21], though at least one case has been reported in a child [22]. Interestingly, this young age of occurrence in our study could argue for a gender difference related to sex hormone status, or for a genetic anomaly—at least in some patients—since in addition 11% of Dercum’s and 27% of LMS cases were apparently familial.

Despite a difference of distribution of lipomas between DD and LMS groups, a very similar metabolic phenotype was found in the two lipomatosis groups, a feature already known in DD, but never reported in LMS. Taking into account the well-known relationship between adipose tissue, insulin resistance and inflammation/immunity, we then considered the immune-hematological phenotype of the two groups of lipomatosis. Despite their similar degree of obesity, they differed by more than twofold higher basophil levels in the Dercum’s group and a significantly lower CD 3^+^ and CD4^+^ & CD8^+^ T lymphocytes subpopulations levels in the LMS group. The next question was then to identify whether those findings were related to obesity [23–26] or if they were specific to the lipomatosis. Therefore, we compared the 2 lipomatosis groups with lean or obese controls.

### Comparison of the Dercum’s disease with the control groups

The Dercum’s group had a metabolic phenotype significantly different from an age-matched lean control group and showed a profile of increased levels of leukocytes and platelets that had already been reported in metabolic syndrome [23–25]. In the present study, this increase was probably related to obesity since this hematological profile did not differ from that one of an age- and sex-matched obese control group. Intriguingly, basophils, which are the least common granulocytes, were also significantly increased in Dercum’s disease compared with lean, but also obese controls, arguing for a specific Dercum abnormality not related to obesity. Basophils have recently been shown to initiate and expand inflammation through the production of specific cytokines and proteases (serine proteases and mast cell protease 8 and 11). Basophil activation is associated with T helper 2 (Th2) immune responses [27, 28] and elicits microvascular hyperpermeability and leukocyte infiltration in affected tissues. Interestingly, basophil infiltrates are a marker of many human cutaneous diseases [29].It is of interest to know, that mast cells are very similar to basophil granulocytes. Basophils leave the bone marrow already mature, whereas the mast cells circulate in an immature form, only maturing once in a tissue site. Both are granulated cells that contain histamine, serotonin and heparin. Increased peripheral serotonin is associated with obesity [30]. Inhibition of peripheral serotonin synthesis and genetic deletion of serotonin synthesis by mast cells have been shown to prevent the development of obesity and insulin resistance, a pathway that need to be further investigated in Dercum’s disease [31, 32].

In addition, a lower number of NK cells was found in Dercum’s disease, significant as compared to a lean control group, and with a trend to significance as compared to an obese group. In mice models, NK cells remove unhealthy adipocytes and stimulate the differentiation of healthy adipocytes. Therefore, a low level of NK cells in adipose tissue could participate in a low level of adipose tissue remodelling, favouring inflammation and insulin resistance [33, 34]. Interestingly, NK cells and basophils are the key cells involved in priming and developing in vivo Th2 responses [35]. The results of the present study then suggest that the underlying mechanisms of Dercum’s disease could be—at least in part—mediated through a low level of NK cells, favouring basophil activation, chronic subclinical inflammation and pain. It is, however, difficult to know if these immune alterations found in blood are also present in adipose tissue and are the cause or the consequence of the metabolic syndrome. Their absence in the Roch-Leri group and in the 2 lean and obese control groups suggests that they might participate in the specific clinical presentation of Dercum’s disease with its recurrent painful lipomas which could correspond to inflammatory foci of adipose tissue.

### Comparison of the Roch-Leri lipomatosis and the control groups

Roch-Leri lipomatosis was associated to the same metabolic phenotype as in the Dercum’s group, significantly different from that one of a lean control group. In contrast, as compared to lean and Dercum groups, Roch-Leri lipomatosis was characterized by a significant decrease of total plasmatic CD3^+^ T cells, and to a lesser extent of CD4^+^ T helper/regulatory T cells and cytotoxic CD8^+^ T cells. A similar profile has already been reported in overweight subjects [26]. Nevertheless, in the present study, the immune profile was significantly different from that one found both in the Dercum’s and the obese groups, arguing for another- perhaps specific- mechanism in Roch-Leri lipomatosis. Interestingly, this group showed a relatively high proportion of patients with autoimmune disease. The association between lymphopenia and autoimmunity is recognized, but the underlying mechanisms are poorly understood [36, 37]*.* A sex-specific adipose tissue imprinting of regulatory T cells has also been shown and could participate to these variations since a predominance of male was found in the LMS group [38]. Nevertheless*,* due to the low number of patients studied, the results need to be regarded with caution, although a low-grade deficit of adaptive immunity could be considered.

Ultimately, this first analysis of Dercum and LMS lipomatosis shows its nearly constant association with obesity and metabolic syndrome, but with different immuno-hematological features. Some of them (increase of blood cell count) seem unspecific findings related to obesity. Others (increase of basophils in DD, decrease of CD4, CD3 and CD8 lymphocytes subpopulations in LMS) seem more specific since significantly different from lean and obese control groups. The QOL (Quality of Life) is impaired in Dercum’s disease, [12], a fact which is confirmed by the high level of use of analgesics and antidepressant drugs in the present study. Therefore, a treatment targeting basophil activation such as omalizumab or benlizumab and perhaps serotonin synthesis [39], could be an option when available.

## Conclusion

In conclusion, Dercum’s disease and Roch-Leri lipomatosis associate multiple lipomas, which are painful in the case of Dercum’s disease, and a metabolic syndrome with obesity, a hallmark which had not been reported for LMS yet. As compared to lean and obese controls, Dercum’s disease is characterized by a twofold increase of basophils associated with a low NK cell count possibly participating to pain. A decreased number of CD3, CD4, and CD8 is present in Roch-Leri lipomatosis. If confirmed, these specific abnormalities could offer new understanding and specific therapeutic opportunities for these patients.

## Supplementary Information

**Additional file 1: Additional figure 1**. Clinico-biological metabolic characteristics of Dercum’s disease (DD) and Roch Leri lipomatosis (LMS) compared with the control group. *:* p* < 0.05, **:* p* < 0.01, ***:* p* < <0.001, ns: non significant;* p* > 0.05. A, B, C, D, E: comparison of clinical characteristics between lipomatosis groups and the control group: A: sex-ratio; B: age (years); C: weight (kilograms); D: BMI (Body Mass Index) (kg/m^2^); E: SBP (Systolic Blood Pressure) (mmHg), F, G, H, I: comparison of biological metabolic characteristics between lipomatosis groups and the control group: F: LDL-c (Low density lipoprotein cholesterol) (g/L); G: FBG (Fasting Blood Glucose)(g/L); H: HOMA-IR (Homeostatic Model Assessment of Insulin Resistance); I: Gamma-GT (Gamma-Glutamyl Transferase) (ui/L), J, K, L: comparison of fat mass markers and distribution between lipomatosis groups and the control group: J: Leptin (ng/mL); K: Fat mass, measured by DEXA (Dual x-ray absorptiometry)(%); L: Intra/Total abdominal fat ratio, measured by MRI (Magnetic Resonance Imaging).

**Additional file 2: Additional figure 2**. Lymphocyte immunophenotyping of Dercum’s disease and Roch Leri lipomatosis (LMS) compared with the control group; ABCDE: comparison of lymphocyte immunophenotype between lipomatosis groups and the control group: A: CD3 +: cluster of differentiation 3; B: CD4 +: cluster of differentiation 4; C: CD8 +: cluster of differentiation 8; D: Lymphocytes: B cells; E: NK: Lymphocytes: Natural Killer cells; F: comparison of basophil lymphocytes between lipomatosis groups and the control groupLymphocyte immunophenotyping of Dercum’s disease and the control group.

## Data Availability

The datasets during and/or analysed during the current study available from the corresponding author on reasonable request.
