# Supplementary material for: Metabolic and immunological phenotype of rare lipomatoses: Dercum’s disease and Roch-Leri mesosomatic lipomatosis
Source: Orphanet J Rare Dis. 2021 Jun 29;16:290. doi: 10.1186/s13023-021-01920-3 (PMC8243498; doi:10.1186/s13023-021-01920-3)
Supplement: Supplementary file 1 — Additional file 1: Additional figure 1. Clinico-biological metabolic characteristics of Dercum’s disease (DD) and Roch Leri lipomatosis (LMS) compared with the control group. *: p < 0.05, **: p < 0.01, ***: p < <0.001, ns: non significant; p > 0.05. A, B, C, D, E: comparison of clinical characteristics between lipomatosis groups and the control group: A: sex-ratio; B: age (years); C: weight (kilograms); D: BMI (Body Mass Index) (kg/m2); E: SBP (Systolic Blood Pressure) (mmHg), F, G, H, I: comparison of biological metabolic characteristics between lipomatosis groups and the control group: F: LDL-c (Low density lipoprotein cholesterol) (g/L); G: FBG (Fasting Blood Glucose)(g/L); H: HOMA-IR (Homeostatic Model Assessment of Insulin Resistance); I: Gamma-GT (Gamma-Glutamyl Transferase) (ui/L), J, K, L: comparison of fat mass markers and distribution between lipomatosis groups and the control group: J: Leptin (ng/mL); K: Fat mass, measured by DEXA (Dual x-ray absorptiometry)(%); L: Intra/Total abdominal fat ratio, measured by MRI (Magnetic Resonance Imaging). [file 13023_2021_1920_MOESM1_ESM.docx]

******

**Figure 2: Clinico-biological metabolic characteristics of Dercum’s disease (DD) and Roch Leri lipomato-sis (LMS) compared with the control group.**

**: p<0.05, **: p<0.01, ***: p<0.001, ns: non significant ; p> 0.05*

*Range*

**A, B, C, D, E:** ***comparison of clinical characteristics between lipomatosis groups and the control group:* A:** sex-ratio***;* B:** age (years)***;* C:** weight (kilograms) ***;* D:** BMI (Body Mass Index) (kg/m^2^)

**E**: SBP (Systolic Blood Pressure) (mmHg)

**F, G, H, I**: ***comparison of biological metabolic characteristics between lipomatosis groups and the control group:*** **F:** LDL-c (Low density lipoprotein cholesterol) (g/L); **G:** FBG (Fasting Blood Glucose) (g/L); **H:** HOMA-IR (Homeostatic Model Assessment of Insulin Resistance) ; **I:** Gamma-GT (Gamma-Glutamyl Transferase) (ui/L)

**J, K, L: *comparison of fat mass markers and distribution between lipomatosis groups and the control group:* J:** Leptin (ng/mL) ***;* K**: Fat mass, measured by DEXA (Dual x-ray absorptiometry)(%) ***;* L:** Intra/Total abdominal fat ratio, measured by MRI (Magnetic Resonance Imaging)
