# Supplementary material for: Metabolic and immunological phenotype of rare lipomatoses: Dercum’s disease and Roch-Leri mesosomatic lipomatosis
Source: Orphanet J Rare Dis. 2021 Jun 29;16:290. doi: 10.1186/s13023-021-01920-3 (PMC8243498; doi:10.1186/s13023-021-01920-3)
Supplement: Supplementary file 2 — Additional file 2: Additional figure 2. Lymphocyte immunophenotyping of Dercum’s disease and Roch Leri lipomatosis (LMS) compared with the control group; ABCDE: comparison of lymphocyte immunophenotype between lipomatosis groups and the control group: A: CD3 +: cluster of differentiation 3; B: CD4 +: cluster of differentiation 4; C: CD8 +: cluster of differentiation 8; D: Lymphocytes: B cells; E: NK: Lymphocytes: Natural Killer cells; F: comparison of basophil lymphocytes between lipomatosis groups and the control groupLymphocyte immunophenotyping of Dercum’s disease and the control group. [file 13023_2021_1920_MOESM2_ESM.docx]

**Figure 3: Lymphocyte immunophenotyping of Dercum’s disease and Roch Leri lipomatosis (LMS) compared with the control group;**

**: p<0.05, **: p<0.01, ***: p<0.001, ns: non significant p> 0.05*

**ABCDE**: ***comparison of lymphocyte immunophenotype between lipomatosis groups and the control group:* A**: CD3 +: cluster of differentiation 3***;* B:** CD4 +: cluster of differentiation 4***;* C**: CD8 +: cluster of differentiation 8***;* D**: Lymphocytes: B cells***;* E**: NK: Lymphocytes: Natural Killer cells

**F:** ***comparison of basophil lymphocytes between lipomatosis groups and the control group***
